# The Evolution of Science in Second Language Acquisition Research: A Commentary on “The Neurocognitive Underpinnings of Second Language Processing: Knowledge Gains From the Past and Future Outlook”

**DOI:** 10.1111/lang.12599

**Published:** 2023-07-25

**Authors:** Viorica Marian

**Affiliations:** Northwestern University

**Keywords:** language learning, SLA, bilingualism, neural networks, science

In 1998, while at a symposium in New York City organized by applied linguist Professor Aneta Pavlenko, I was introduced to one of the other panelists whose last name started with “Van” and who turned out to be from the same small village in the Netherlands as my Dutch in-laws. That day, a professional camaraderie was born, one that saw us reconnecting at conferences and meetings in many countries and universities over the years. It saw us start faculty positions, establish and run laboratories, design experiments, write papers, teach and mentor students, earn tenure, then promotion, then even more service. It also saw us raise children, cope with aging parents, and navigate life. Decades summed in one paragraph, because academic journals leave out the intertwined work and life challenges of the professoriate. And yet, academia is punctuated by precisely this type of professional relationships built over decades of conference conversations in the pursuit of knowledge—the short-term ontogeny of individual lives evolving alongside the long-term phylogeny of science.

Science can seem relentless at times—there are always more questions to be answered, and asked, new things to be discovered, knowledge to be gained. Especially when it comes to a field as young as neuroscience, where new tools and methodologies drive discoveries every day. It can be exciting to figure out the answers to questions that nobody in the world knows yet. In the case of second language (L2) learning and processing, these are questions about how the brain learns and manages two or more languages.

Professor Janet van Hell’s contributions to understanding the neurocognitive correlates of L2 learning and processing have helped shape the field over the years. Her keynote article has offered readers a comprehensive review of the neuroscience of L2 processing, starting with the basics of electroencephalography and functional magnetic resonance imaging technologies through the many variables that impact L2 neural, structural, and functional changes, all the way to the neural networks that support language processing in multilingual speakers, as well as current challenges and future directions. It has provided a concise overview of the literature and is an excellent introduction to the field for newcomers who want to understand its evolution.

Although in science at large the monolingual prism has continued to dominate, those who study language are acutely aware of the diversity of language experiences around the world and know that language experience shapes the brain. The studies discussed in this review have clearly shown that as language experiences change, so do the neural networks subserving them. The networks activated by language are not identical across speakers with different language backgrounds and indeed not even within speakers as their language experiences evolve.

Moment by moment, individual experiences, including language experiences, continually rewrite one’s neural networks (Marian, 2023). The variability in language use is tied to variability in neural networks. As language knowledge advances, as new words and grammars and functions are acquired, the connectivity of the neural network is modified. It has been proposed that the constant juggling of two or more languages creates a more interconnected neural network that is responsible for the cognitive and neural reserve observed in speakers of multiple languages.

Experimental evidence has suggested that using multiple languages changes the neural processing of speech in children (Krizman et al., 2015) and the neural consistency and attentional control of adolescents (Krizman et al., 2014). In adults, experience with multiple languages changes the neural signatures of language learning (Bartolotti et al., 2017), the cortical control of between- and within-language competition (Marian et al., 2017), and the recruitment of executive control regions during phonological competition (Marian et al., 2014). Beyond cortical changes, experience with L2 processing also modifies the subcortical encoding of sound (Krizman et al., 2012). Language experience even impacts biological phenomena such as otoacoustic emissions, which are sounds generated from within the inner ear (Marian et al., 2018).

Indeed, there is a rich body of evidence from empirical research conducted in laboratories around the world that has yielded support for Dr. van Hell’s review of the neurocognitive underpinnings of L2 processing. I particularly appreciated that the article highlighted recent findings that the degree of functional connectivity within the language network is shaped by the developmental timeline of L2 learning. Also notable, the review did not miss the need for increased diversity in neurocognitive research, the limitations of averaging across individual differences, and the acknowledgment of the many other yet-unanswered puzzles waiting to be solved. The science of L2 acquisition has evolved from a history of case studies and descriptive analyses to empirical cognitive and behavioral experiments, to current neuroscientific investigations, to a future of large data and human-machine interaction.

I believe that, at the present time, the neural network approach is a particularly productive direction to pursue in the neuroscience of bilingualism, despite the admittedly still-limited tools at researchers’ disposal. The review mentioned recent work on the language network, the default-mode network, and resting-state functional connectivity in those who know more than one language. The neural network approach to studying L2 processing is particularly relevant in today’s emerging world of large language models, with generative pretrained transformers taking center stage. These models challenge many long-held assumptions in the field of language learning, from innate grammar to critical age, and raise questions about similarities and differences between how humans and machines learn language and even about the definition of language itself.

The current discussions about large language models and Dr. van Hell’s review of the neurocognition of L2 processing suggest that the field of L2 learning and processing is entering an era in which the neurocognitive underpinnings of language will gain increased relevance. As the AI race heats up, researchers must make sure that questions about L2 processing, bilingual and multilingual experience, and linguistic diversity are an integral part of those conversations (Marian, 2023).
